# Supercritical Carbon Dioxide Extraction of Seed Oil from Winter Melon (*Benincasa hispida*) and Its Antioxidant Activity and Fatty Acid Composition

**DOI:** 10.3390/molecules18010997

**Published:** 2013-01-15

**Authors:** Mandana Bimakr, Russly Abdul Rahman, Farah Saleena Taip, Noranizan Mohd Adzahan, Md. Zaidul Islam Sarker, Ali Ganjloo

**Affiliations:** 1 Department of Food Technology, Faculty of Food Science and Technology, Universiti Putra Malaysia, Serdang 43400, Selangor, Malaysia; E-Mails: mandanabimakr@yahoo.com (M.B.); noraadzahan@food.upm.edu.my (N.M.A.); aganjloo@yahoo.com (A.G.); 2 Department of Process and Food Engineering, Faculty of Engineering, Universiti Putra Malaysia, Serdang 43400, Selangor, Malaysia; E-Mail: saleena@eng.upm.edu.my; 3 Halal Product Research Institute, Universiti Putra Malaysia, Serdang 43400, Selangor, Malaysia; 4 Department of Pharmaceutical Technology, Faculty of Pharmacy, International Islamic Universiti Malaysia, Kuantan 25200, Pahang, Malaysia; E-Mail: zaidul@iium.edu.my; 5 Department of Food Science and Technology, Faculty of Agriculture, Zanjan University, Zanjan P.O. Box 313, Iran

**Keywords:** winter melon (*Benincasa hispida*), supercritical carbon dioxide extraction, response surface methodology, fatty acid composition, antioxidant activity

## Abstract

In the present study, supercritical carbon dioxide (SC-CO_2_) extraction of seed oil from winter melon (*Benincasa hispida*) was investigated. The effects of process variables namely pressure (150–300 bar), temperature (40–50 °C) and dynamic extraction time (60–120 min) on crude extraction yield (CEY) were studied through response surface methodology (RSM). The SC-CO_2_ extraction process was modified using ethanol (99.9%) as co-solvent. Perturbation plot revealed the significant effect of all process variables on the CEY. A central composite design (CCD) was used to optimize the process conditions to achieve maximum CEY. The optimum conditions were 244 bar pressure, 46 °C temperature and 97 min dynamic extraction time. Under these optimal conditions, the CEY was predicted to be 176.30 mg-extract/g-dried sample. The validation experiment results agreed with the predicted value. The antioxidant activity and fatty acid composition of crude oil obtained under optimized conditions were determined and compared with published results using Soxhlet extraction (SE) and ultrasound assisted extraction (UAE). It was found that the antioxidant activity of the extract obtained by SC-CO_2_ extraction was strongly higher than those obtained by SE and UAE. Identification of fatty acid composition using gas chromatography (GC) showed that all the extracts were rich in unsaturated fatty acids with the most being linoleic acid. In contrast, the amount of saturated fatty acids extracted by SE was higher than that extracted under optimized SC-CO_2_ extraction conditions.

## 1. Introduction

Free radicals caused by stress, illness, drugs and pollution induce damage to cell structures, DNA, lipids and proteins also play a key role in cancer, atherosclerosis, cardiovascular disease and neurological disorders [1,2]. Antioxidants are able to protect human body against damages of free radicals due to their redox properties [3]. Natural antioxidants have received considerable attention due to the toxic effects of synthetic antioxidants including propyl gallate (PG), butylated hydroxytoluene (BHT) and butylated hydroxyanisole (BHA) on human health [4]. It was revealed that herbs, seeds and spices are potential sources of natural antioxidants due to their content of phytochemicals such as tannins, diterpenes, flavonoids and phenolic acids [5].

*Benicasa hispida* L., a member of the family named Cucurbitaceae, is a plant originated from south-east Asia and cultivated for at least 2,000 years [6]. *Benincasa hispida* L. is variously named winter melon, white gourd, ash pumpkin, tallow gourd, white pumpkin, ash gourd, wax gourd, gourd melon and Chinese watermelon or Chinese preserving melon in English [7]. Depending on the shape, type and maturity of the fruit, the seeds, which are smooth and white to yellowish-colored, fill the centre of the fruit [6,8]. Index of Nutritional Quality (INQ) data shows that *Benincasa hispida* is valued as a high quality vegetable [6]. Several investigations have previously focused on the biologically active components of *Benincasa* species and its antioxidant activity on different tissues like liver and brain has been proven [9]. Therefore, preparing extract rich in bioactive compounds is essential for industrial utilization of *Benincasa hispida* seeds.

Extraction is a major step for the isolation, identification and use of valuable compounds from different plants [10]. Numerous studies were carried out for the development of novel extraction processes to augment the product quality and the quantity of the active natural products [11,12]. In recent years, supercritical fluid extraction (SFE) has received considerable attention as a promising alternative to conventional technology for separation of different valuable compounds from natural sources [13,14]. This is due to the fact that this technique is usually performed at low temperatures, with short extraction times and a small amount of solvent compared to conventional extraction methods which have been used to extract compounds for a long time, involving usage of a large amount of solvents and high temperatures [15].

Supercritical carbon dioxide (SC-CO_2_) extraction has attracted a lot of attention due to the fact that carbon dioxide (CO_2_) is an inert, inexpensive, non-toxic and environmentally-friendly solvent which allows extraction at low temperatures and relatively low pressures. In addition, CO_2_ can be immediately evaporated when exposed to atmospheric conditions, thus, the extract obtained is free from chemicals and thermal degradation compounds [16]. In fact, SC-CO_2_ extracts are generally recognized as safe (GRAS) to be used in food products [17]. Furthermore, CO_2_ is a non polar solvent therefore, adding a small amount of polar solvents as co-solvent can enhance greatly the extraction efficiency of polar compounds. Among different solvents ethanol (EtOH) is the most used solvent due to its good miscibility with CO_2_, non-toxicity and allowed use in the food and pharmaceutical industries [16]. Previously, SC-CO_2_ has been used to extract valuable compounds from different raw materials such as hazelnut [18], grape seed [19], watermelon [20], orange pomace [21], peach (*Prunus persica*) seed oil [22], *Bidens pilosa* Linné [23] and *Mitragyna speciosa* leaves [24]. Herrero *et al.* [25] presented an overview on the recent advances and applications of SFE. Furthermore, SFE has been proposed for separation of antioxidant compounds from sage, rosemary leaves and some Brazilian plants [26,27,28], but to the best of our knowledge no extraction of *Benincasa hispida* by SC-CO_2_ has been reported.

Optimization of the experimental conditions is a critical step to develop a successful process due to the effect of various variables including pressure, temperature and extraction time on the efficiency of SC-CO_2_ extraction. Over the past several years, response surface methodology (RSM) has been used in many areas such as foods, chemicals, or biological processes. This experimental methodology combines mathematics with statistics for generating a mathematical model to describe process, examine the relationship between one or more response variables and a set of quantitative experimental variables and optimizing the process, while at the same time reducing the number of experimental trials [14,29]. Nowadays, RSM is widely used to optimize the conditions of SFE method [11,14]. The objectives of this study were to evaluate the effect process variables including pressure, temperature and dynamic extraction time on crude extract yield (CEY). RSM was employed to optimize extraction conditions in order to obtain the maximum CEY. Furthermore, the antioxidant activity and fatty acid composition of the extract obtained under optimized conditions were determined and then compared with those obtained by the Soxhlet and UAE method.

## 2. Results and Discussion

### 2.1. Effect of the Process Variables on Crude Extraction Yield

**Figure 1:**
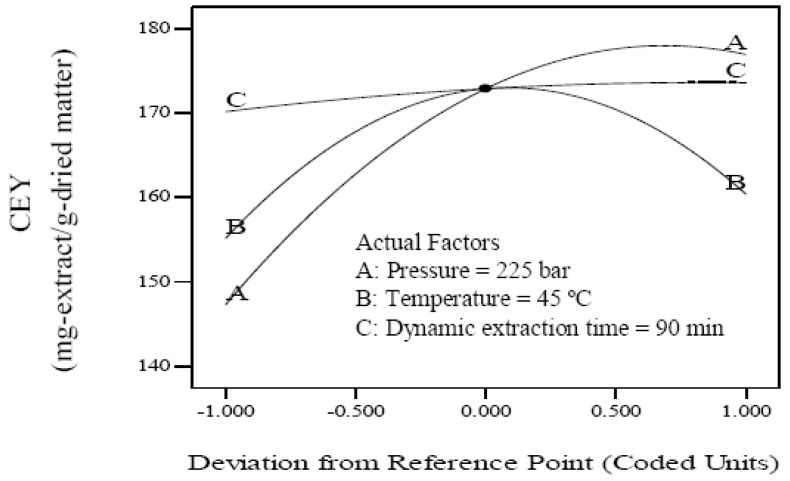
Perturbation graph showing the effect of process variables on crude extract yield.

The effects of pressure, temperature and dynamic extraction time on the CEY were evaluated. The individual effect of independent variables including pressure (A), temperature (B) and dynamic extraction time (C) was found by perturbation plot. A perturbation plot does not show the effect of interactions and it is like one factor-at-a-time experimentation. The perturbation plot helps to compare the effect of all independent variables at a particular point in the design space. The response is plotted by changing only one factor over its range while holding the other factors constant. A steep slope or curvature in a factor shows that the response is sensitive to that factor. A relatively flat line shows insensitivity to change in that particular factor [30]. Therefore, it was revealed that CEY is more sensitive to pressure and temperature than dynamic extraction time. The perturbation plot for the CEY is shown in Figure 1.

### 2.2. Optimization of SC-CO_2_ Extraction Conditions and Verification of the Model

The experimental design and related results are presented in Table 1. The CEY ranged from 119.11 to 173.33 mg-extract/g-dried sample. A second-order polynomial model for predicting CEY was rendered by multiple linear regression analysis of the experimental data. Estimated regression equation coefficients and statistical significance of linear, quadratic and interaction of process variables along with the corresponding R^2^, adjusted-R^2^ and lack of fit test for the final reduced model are presented in Table 2. Probability values (*p*-value) revealed that all linear, quadratic and interaction terms had significant (*p* < 0.05) effect on the CEY. The *p*-value of final reduced model was less that 0.05 which confirmed that model fitness was significant (Table 2). Furthermore, the lack of fit was non-significant (*p* > 0.05), indicating that the model could adequately fit the experiment. The developed final reduced model in term of CEY was obtained as follows:

CEY=172.87+14.82X_1_+2.61X_2_+1.72X_3_−10.77X_1_^2^−15.11X_2_^2^−0.99X_3_^2^−2.51X_1_X_2_−0.88X_1_X_3_−0.90X_2_X_3_ (1)

where X_1_, X_2_ and X_3_ are the coded variables for pressure, temperature and dynamic extraction time, respectively.

**Figure 2:**
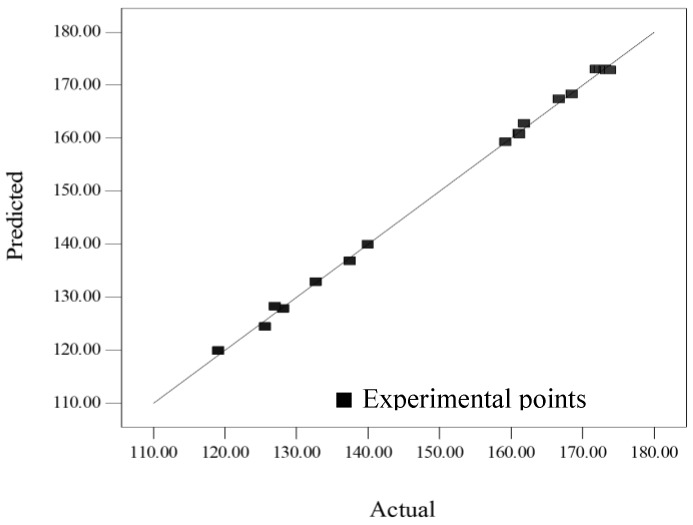
Plot of predicted crude extraction yield related with experimental values.

Figure 2 indicates that the predicted values were very close to the actual values. Indeed, the high value of coefficient of determination, *R*^2^ (0.998) and adjusted-*R*^2^ (0.997) confirmed the adequate accuracy of the polynomial reduced model. The small E value (0.35%) suggested that the obtained model was acceptable. Moreover, the coefficient of variation (C.V.% = 0.630) indicated that the final reduced model was reproducible.

**Figure 3:**
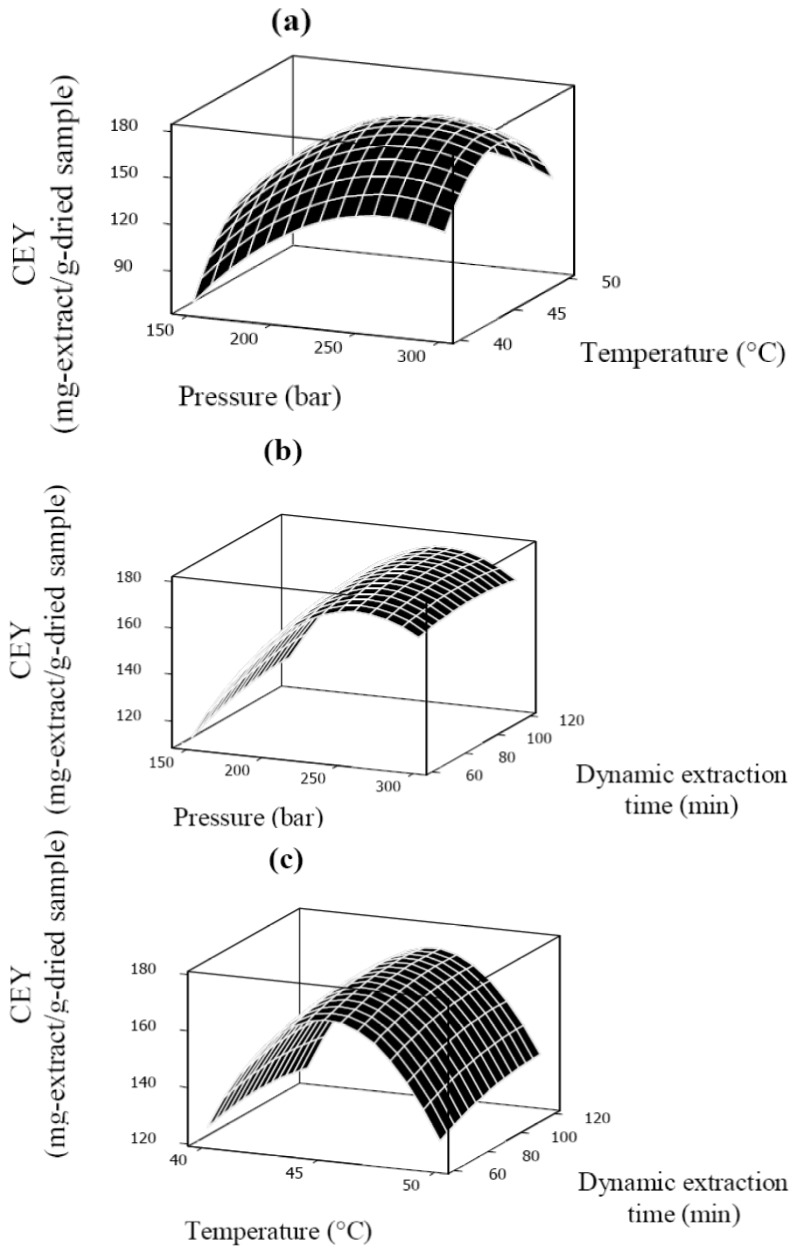
Response surface plots for CEY (mg-extract/g-dried sample) as a function of: (**a**) pressure (bar) and temperature (°C), (**b**) pressure (bar) and dynamic extraction time (min), (**c**) temperature (°C) and dynamic extraction time (min).

The best way to visualize the influence of the process variables on the response is to generate three dimensional surface response plots [30]. Response surface plots are illustrated in Figure 3a–c. Figure 3a illustrates the effect of pressure and temperature on the CEY at a fixed dynamic extraction time of 90 min. It is clear that there is an optimal value for pressure to obtain the highest CEY. At a fixed temperature level, the CEY increased with increasing pressure up to a certain value (around 250 bar), which was due to the increased SC-CO_2_ density at higher pressure [15]. It was known that as pressure increased the extraction rate also increased due to the changes in solubility of solutes in solvent [18,19,20,21,22,23,24]. However, in the current study, further increase in the pressure level (above 250 bar) led to an unexpected reduction in the CEY. Same unexpected behavior was also observed by Rezaei and Temelli [31] which they stated it could be related to the reduced diffusion rates of the solutes from the plant matrix to the SC-CO_2_ medium. Another possible explanation for this unexpected reduction could be compressing of seeds at higher pressure. Luengthanaphol *et al*. [32] reported that increasing pressure compresses the tamarind seed coats and caused reducing ability for solvent used to diffuse inside the seed particles.

The solubility of the solute is likely to decrease, keep constant, or increase with the temperature rise at constant pressure, which depends on the solvent density and the solute vapor pressure [33,34,35,36,37]. As presented in Figure 3a,c, an increase in CEY was observed with enhancing of the temperature in an early stage of extraction, because the change of solvent density is less effective than that of solute vapor pressure, but the trend was reversed when the temperature reached a certain value under the given range of pressure range for this work.

Similar results were also reported for the extraction of tea (*Camellia sinensis* L.) seed oil [34] and extraction of lipids by SC-CO_2_ extraction from grain sorghum [38]. Since the solubility of the solutes depends on the balance between fluid density and solute vapor pressure, both controlled by fluid pressure and temperature, an extraction temperature and pressure in the range from 40 to 47 °C, 150 bar to 270 bar, respectively, is practical during the SC-CO_2_ extraction of *Benincasa hispida* seed oil.

Dynamic extraction time had a positive effect on the CEY of *Benincasa hispida* seed oil. As shown in Figure 3b,c the CEY was increased with dynamic extraction time. It was clear that there is an optimal level for the dynamic extraction time. It should be mentioned that due to the physical structure of the seeds, the penetration of the solvent and the diffusion of the unreleased oil in the particles are very slow. Therefore, dynamic extraction time is limited to the fast extraction period since the amount of oil recovered in the slow extraction period is negligible. However, the duration of fast extraction period is affected by temperature and pressure. In this study, above the moderated extraction pressure (225 bar) and temperature (45 °C), by increasing dynamic extraction time beyond 100 min the CEY did not changes significantly (*p* > 0.05). Same result was found by Xu *et al.* [30] investigating the effect of extraction time on SC-CO_2_ extraction of sea buckthorn (*Hippophae thamnoides* L.) oil.

The regression equation obtained in this study was used to maximize the CEY from *Benincasa hispida* seed as the desired optimum condition of the extraction process. In this regard, a numerical optimization was performed through desirability function method. The optimal conditions for the desired goal were predicted as 244 bar pressure, 46 °C temperature and 97 min dynamic extraction time. The SC-CO_2_ extraction of *Benincasa hispida* seed under the optimal conditions was performed in triplicate as a rechecking experiment and then the obtained value were compared with the predicted result. The mean value of 175.60 mg-extract/g-dried sample obtained from the real experiment was very close to the predicted value (176.30 mg-extract/g-dried sample). This finding indicates the validity and adequacy of the rendered model to reflect the expected optimization.

### 2.3. Antioxidant Activity of Extracted Crude Oil

**Figure 4:**
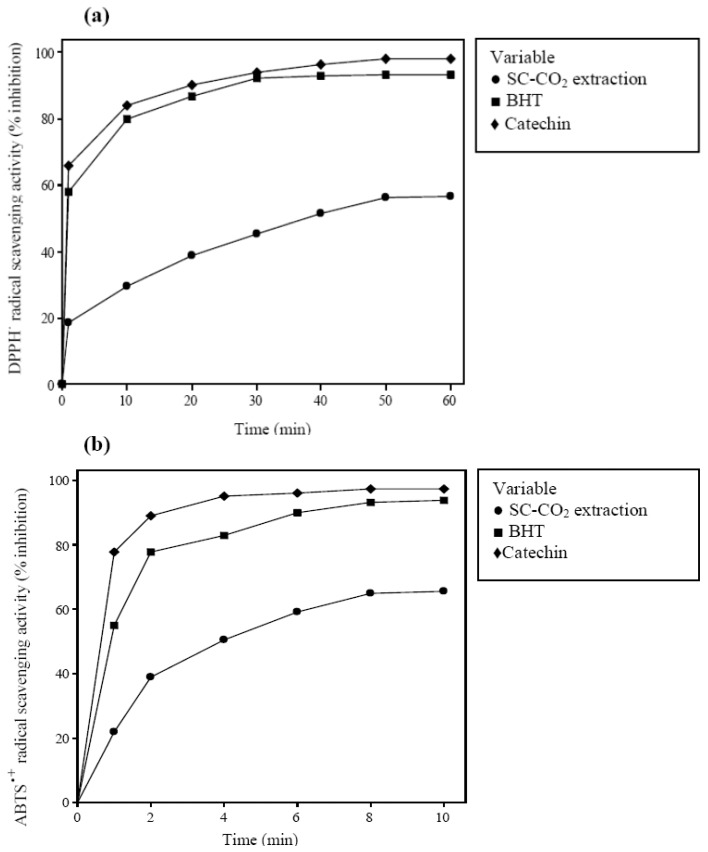
Antioxidant activity of extract determined by (**a**) DPPH**˙** radical scavenging assay (**b**) ABTS**˙^+^** radical scavenging assay (standard deviation bars are smaller than the symbol size).

The antioxidant activity of *Benincasa hispida* seed oil was determined through the DPPH**˙** and ABTS**˙^+^** radicals scavenging assays. The results obtained under optimized condition of SC-CO_2_ extraction compared with the antioxidant activity of catechin and BHT as a synthetic antioxidant Figure 4a,b. The antioxidant activity of *Benincasa hispida* seeds extract was lower compared with those of synthetic antioxidants at the same concentration (0.1 mg/mL). As reported by Liu *et al*. [34] the antioxidant activity values for seeds oil from *Opuntia dillenii* Haw ranged from 90 to 17% when the concentrations varied from 4 to 0.1 mg/mL, accordingly. Therefore, it was expected that this weak point could be addressed by using higher concentration of extracts which is of great interest and need to be further investigated.

### 2.4. Identification and Quantification of Fatty Acid Composition

The fatty acid composition of a kind of extracts is its most useful chemical feature. Many of the chemical tests for extracts identity or purity can be related to their fatty acid content [34]. The fatty acid composition of extract obtained using optimized SC-CO_2_ extraction is presented in Table 3. The total saturated fatty acid (SFA) content (myristic acid, palmitic acid and stearic acid) was 15.97% of total fatty acid, and the palmitic acid (9.83 ± 0.33% of total fatty acid) content was higher comparatively. However, the total unsaturated fatty acid (UFA) (palmitoleic acid, oleic acid, linoleic acid and linolenic acid) content was 83.53% of total fatty acids. The major UFA was linoleic acid (18:2) which constitutes 67.17% of total fatty acid. α-Linolenic acid, which is a kind of omega-3 fatty acid, was detected in extract obtained by the optimized SC-CO_2_ extraction method. The presence of essential fatty acids (EFAs), namely linoleic acid (C18:2) and α-linolenic acid (C18:3) in *Benincasa hispida* seed oil demonstrated its importance in the human body. These two EFAs are necessary to support normal cell functions, promote disease prevention and may even be useful for disease treatment [39].

### 2.5. Comparison with Other Extraction Techniques

Different extraction processes have different extraction yield and efficiencies. Higher concentration of valuable compounds in the extracts is an important aspect in the production of natural products while a primary task in the industries is lowering economic cost which can be achieved by better extraction yield [11]. As shown in Table 3, the results of this study were compared with those obtained and published with UAE [40] and SE [41]. According to the results, SE resulted in the highest value of CEY (250.00 ± 1.30 mg-extract/g-dried sample) comparing to the UAE and optimized SC-CO_2_ extraction. The efficiency of UAE and optimized SC-CO_2_ extraction techniques for recovery of CEY from *Benincasa hispida* seeds was 43% and 70% of those found with SE, respectively, but SC-CO_2_ extracts were found to possess better quality in terms of antioxidant activity compared to those obtained with UAE and SE processes.

Natural bioactive compounds which are contributed to the antioxidant activity of extracts are thermo sensitive and may decompose at higher temperature (78 °C) for long extraction time (6 h) which was applied for SE. UAE is one of the simplest extraction techniques because it is easy to operate in common laboratory equipment but its efficiency in terms of CEY was lower than SE and optimized SC-CO_2_ extraction. SC-CO_2_ extraction has different advantages over SE such as low operating temperature, thus no thermal degradation of most of the labile compounds, shorter extraction duration and high selectivity in the extraction of target compounds. SC-CO_2_ extraction also, seems to be a cost-effective process at laboratory scale, but a precise economic evaluation will need additional experiments for establishing large-scale units [42].

As shown in Table 3 the fatty acid contents of the extracts from different extraction methods (SE, UAE and SC-CO_2_) were different, however, the difference between UAE and SC-CO_2_ extracts was minor. The SFAs extracted by SE (24.3% of total fatty acid) was higher than those extracted by optimized SC-CO_2_ extraction (15.97% of total fatty acids) and UAE (17.80% of total fatty acids). In a study which conducted by Rajaei *et al*. [43] they obtained same results about the SC-CO_2_ extraction of tea seeds. They found that the UFAs extracted by SC-CO_2_ extraction were higher than SE. They pointed out that higher temperatures appear to be unfavorable to the extraction of UFAs. It was obtained that in all extracts obtained with different extraction process the linoleic acid (C18:2) was dominant (around 60.60–67.17% of total fatty acids). Furthermore, essential fatty acids (including 18:2 and 18:3) contributed to high percentage (around 68%) of total fatty acids which confirmed the importance of the extracts.

## 3. Experimental

### 3.1. Material and Reagents

Whole winter melons (*Benincasa hispida* L.) were purchased from a local market in Serdang, Selangor, Malaysia. Fruits were chosen at commercial maturity according to their similarity of color, size and absence of surface defects. The fruits were cut, and the seeds were separated manually and washed under tap water. Seeds were dried at 40 °C in a ventilated oven (1350FX, Cornelius, OR, USA) for 24 h and then stored at an ambient temperature in the dark. The seeds were ground in a grinder mill (MX-335, Panasonic, Shah Alam, Malaysia) for 10 s to produce a powder with an approximate size of 1.5–2.5 mm.

Carbon dioxide (CO_2_, SFE grade) contained in a dip tube cylinder was purchased from MOX-Linde Gases Sdn. Bhd. (Petaling Jaya, Malaysia). Analytical grade ethanol and hexane were obtained from Scharlau (Port Adelaide, Australia). Sodium methoxide, potassium persulphate, catechin, 2,2′-azinobis(3-ethylbenzothiazoline-6-sulphonic acid) diammonium salt (ABTS**˙^+^**), 1,1-diphenyl-2-picrylhydrazyl (DPPH**˙**), gallic acid and Folin-Ciocalteu reagent (FCR) were purchased from Fisher (Pittsburgh, PA, USA). Fatty acid methyl ester (FAME) standards were obtained from Sigma-Aldrich (St. Louis, MO, USA). All chemicals were either of chromatography or analytical grade.

### 3.2. Supercritical Carbon Dioxide Extraction

**Figure 5:**
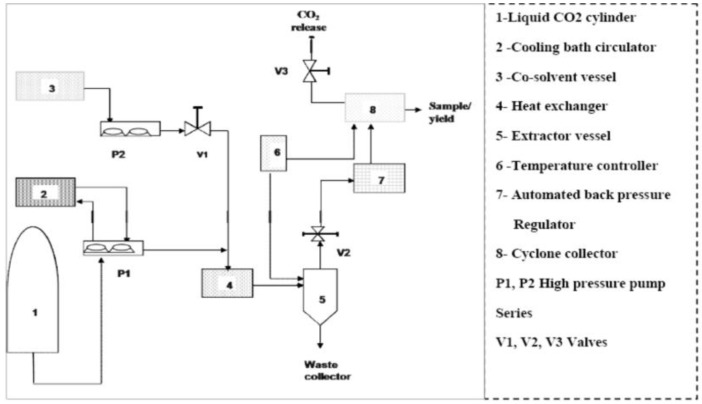
Scheme of experimental set-up for supercritical fluid extractor.

Supercritical carbon dioxide (SC-CO_2_) extraction was carried out in a batch system using a supercritical fluid apparatus (ABRP200, Pittsburgh, PA, USA) including 500 mL extraction vessel, automated back pressure regulator (model BPR-A-200B), high pressure pump (model P-50). Figure 5 displayed a scheme of the supercritical fluid extractor (500 mL sample capacity). The flow rate of CO_2_, the extraction temperature and pressure were adjusted by the ICE software, and the extraction time was measured by a stopwatch. Samples of ground *Benincasa hispida* seeds (40 g) and glass beads (120 g with 2.0 mm in diameter) were mixed and then put into an extraction vessel. The application of some rigid materials such as glass bead with the ground sample is useful for maintaining a proper CO_2_ flow rate in the extraction vessel and maintained the desired permissibility of the particles during extraction process [15]. Before pressurization, the SFE system was allowed to reach the desire operating temperature. Then the liquefied CO_2_ was transferred to the extraction vessel by a high pressure pump (P-50, Thar Designs, Inc., Pittsburgh, PA, USA). In this work, the pressure, temperature and dynamic extraction time of the SC-CO_2_ extraction were varied from 150 to 300 bar, from 40–50 °C and from 60 to 120 min, respectively. The supercritical CO_2_ flow rate was maintained at 10 g/min based on preliminary experiments. A higher yield can be obtained by adding small amount of co-solvent. In all experimental conditions, absolute ethanol (EtOH) was applied as the co-solvent due to its good miscibility with CO_2_, non-toxicity and allowed use in the food and pharmaceutical industries. The co-solvent flow rate was maintained at 1 g/min. After reaching the desired pressure and temperature, the *Benincasa hispida* seeds soaked in the solvent and co-solvent for 30 min (static extraction) in order to equilibrate the mixture at desire pressure and temperature. The static extraction time was applied at desire pressure and temperature for each conducted run. During the dynamic extraction time, CO_2_ carrying the released solutes flowed out of the unit and the extract was collected into a pre-weighed collection flask. The procedure was performed in duplicate.

### 3.3. Crude Extraction Yield Measurement

The extracts were weighed gravimetrically using a Mettler Toledo analytical balance (±0.0001 g) (Mettler Toledo GmbH, Greinfensee, Switzerland) and then the CEY was calculated according to the following equation:

(2) $$CEY=\frac{m_{e}}{m_{s}}\times 1000$$

where m_e_ is the crude extract mass (g) and m_s_ is the extracted sample mass (g). The measurement was performed in triplicate and the mean values of CEY were expressed as mg-extract/g-dried sample.

### 3.4. Determination of Radical Scavenging Activity

The extracts *of Benincasa hispida* seeds were subjected to antioxidant activity analysis using DPPH**˙** and ABTS**˙**^+^ free radical scavenging assays. All determinations were done in triplicate and expressed as means ± Standard Deviation.

#### 3.4.1. Determination of DPPH**˙** Radical Scavenging Activity

This assay was carried out as described by Zengin *et al.* [44] with some modifications. A total of 0.1 mg/mL of the extracts and synthetic antioxidant (catechin) in the ethanol were added into an ethanolic solution of DPPH**˙** (3 mL, 6 × 10^−5^ M). The mixture was vortexed for 20 s at room temperature. Absorbance measurements at 515 nm commenced immediately in a 1 cm quartz cell after 1 min up to 60 min with 10 min intervals using a UV-260 visible recording spectrophotometer (Thermo 4001/4 UV–Vis Spectrophotometer, Thermo Fisher Scientific, West Palm Beach, FL, USA). The blank test was conducted with 0.1 mL ethanol instead of extracts and the absorbance was recorded as A_blank_. The inhibition percent of DPPH**˙** which was scavenged (% DPPH_sc_) was calculated according to the following equation:

% DPPH_sc_ = 100 × (A_blank_ − A_sample_)/A_blank_ (3)

where A_blank_ and A_sample_ are the absorbance values of the blank and of the tested samples, respectively.

#### 3.4.2. Determination of ABTS**˙^+^** Radical Scavenging Activity 

The 2,2′-azinobis(3-ethylbenzothiazoline-6-sulphonic acid) diammonium salt (ABTS**˙^+^**) assay was carried out according to the method of Cai *et al.* [45]. The ABTS**˙^+^** radical solution was prepared by mixing 7 mM ABTS and 2.45 mM potassium persulphate, and incubating the mixture in the dark at room temperature for 16 h. The ABTS**˙^+^** solution was then diluted with 80% (v/v) ethanol to obtain an absorbance of 0.70 at 734 nm. ABTS**˙^+^** solution (3.9 mL) was added to sample (0.1 mg/mL) and mixed vigorously. The absorbance of the mixtures at room temperature was recorded immediately using UV-260 visible recording spectrophotometer (Thermo 4001/4 UV-Vis Spectrophotometer) at 734 nm for 10 min at 2 min intervals. The blank test was conducted with ethanol instead of extracts and the absorbance was recorded as A_blank_. The inhibition percent of ABTS**˙^+^** which was scavenged (%ABTS_sc_) was calculated using the following equation:

%ABTS_sc_ = (A_blank_ − A_sample_) × 100/A_blank_ (4)

where A_blank_ and A_sample_ are the absorbance values of the blank and of the tested samples, respectively.

### 3.5. Preparation of Fatty Acid Methyl Esters 

Samples were brought to a temperature of 50–60 °C and homogenized thoroughly before taking a test sample in order to obtain the fatty acid methyl esters (FAMEs). An aliquot of the test sample (100 µL) was mixed with hexane (1 mL) in a 2 mL vial. An aliquot of sodium methoxide (1 µL, 1% w/v) was added to the vial which was mixed vigorously using a vortex mixer. The mixture first became clear and then turbid as sodium glyceroxide was precipitated. After a few minutes, the clear upper layer of methyl ester was pipetted off and injected into the gas chromatograph (GC) for further analysis. 

### 3.6. Gas Chromatography Analysis

Fatty acids composition analysis was performed in a Hewlett-Packard 6890 gas chromatograph (Wilmington, DE, USA), equipped with a flame ionization detector (FID) and a BPX70 (30 m × 0.25 mm × 0.25 µm, Victoria, Australia) GC column. Oven temperature was programmed isothermally to 115 °C during 2 min, then was raised at 4 °C/min to 163 °C and then at 1 °C/min to 170 °C. Finally, temperature increased to 200 °C at 10 °C/min and held at this temperature for 2 min. Helium was used as a carrier gas which flowed at a rate of 1 mL/min. The injection volume was 1 µL. Standard methyl esters of fatty acids were used as authentic samples. The fatty acids determination was accomplished by comparing it with standards and was valued by the area percentage of each fatty acid. The fatty acid determination was performed in triplicate for each sample and expressed as means ± Standard Deviation.

### 3.7. Experimental Design and Statistical Analysis

Response surface methodology (RSM) was applied to optimize the process variables including pressure (150–300 bar), temperature (40–50 °C) and dynamic extraction time (60–120 min) to achieve the highest amount of crude oil from *Benincasa hispida* seeds. A central composite design (CCD) with axial points was used for designing the experimental data. This generated 20 treatments with six replications at the centre points to estimate the repeatability of the method (Table 1). The effect of unexplained variability induced by extraneous factors on the observed responses was minimized by randomizing the order of experiments. Blocks are assumed to have no impact on the nature and shape of the response surface. The following second-order polynomial model was fitted to the data:

Yi = β_0_ + β_1_X_1_ + β_2_X_2_ + β_3_X_3_ + β_11_X_1_^2^ + β_22_X^2^_2_ + β_33_X_3_^2^ + β_12_X_1_X_2_ + β_13_X_1_X_3_ + β_23_X_2_X_3_

where Y_i_ is predicted response, β_0_ is offset term, β_1_, β_2_ and β_3_ are the regression coefficients for linear effect terms, β_11_, β_22_ and β_33_ are quadratic effects and β_12_, β_13_ and β_23_ are interaction effects. In this model, X_1_, X_2_ and X_3_ represent power level, temperature and sonication time, respectively. The significant terms (*p* < 0.05) in the model were found by analysis of variance (ANOVA) based on *p*-value. The terms statistically found non-significant (*p* > 0.05) were dropped from the initial model and the experimental data was refitted only to significant (*p* < 0.05) variables in order to obtain the final reduced model [46]. 

The three-dimensional response surface plot was generated for the graphical interpretation of the interaction effect of independent variables on the response. Numerical optimization was carried out to predict the exact optimum level of independent variables leading to the desirable response goal. The model adequacy was determined using model analysis, lack of fit test, coefficient of determination (R^2^) and adjusted-R^2^. Furthermore, experimental data were compared with predicted values (method validation) in order to verify the adequacy of final reduced model. In addition, the quality of fit between the experimental and predicted data was determined according to value of the mean relative deviation modulus (E). The criteria can be calculated as follows:

(6) $$E\left(\%\right)=\frac{100}{n}\sum_{i=1}^{n}\frac{\left|V_{exp}-V_{pre}\right|}{V_{exp}}$$

where V_exp_ and V_pre_ are the experimental and predicted values, respectively, n is the number of experimental data. A model is considered acceptable if E value is less than 10% [47]. The experimental design matrix, data analysis, regression coefficients, generation of 3D graph and numerical optimization procedure were created using Design Expert Version 8.0.7 software (Stat-Ease Inc., Minneapolis, MA, USA, trial version).

## 4. Conclusions

In this study, *Benincasa hispida* seed oil has been extracted using SC-CO_2_ under various operating conditions. The effects of pressure, temperature and dynamic extraction time on the crude extraction yield were studied. It was found that all process variables had positive significant effect on the response follows the order of pressure > temperature > dynamic extraction time. Response surface methodology with a central composite design (CCD) was successfully applied for optimization of SC-CO_2_ extraction process. The empirical polynomial equation has been proposed to predict optimum operating conditions of process. The highest crude extraction yield of 175.60 (mg-extract/g-dried sample) was obtained when the process was carried out at 244 bar of pressure, 46 °C of temperature and dynamic extraction time of 97 min. Under these optimized conditions, the experimental crude extraction yield agreed closely with the predicted yield. The antioxidant activities of seed oil obtained by optimized SC-CO_2_ extraction was compared with those obtained and published using SE and UEA techniques. It was found that SC-CO_2_ extract was substantially better than extract obtained by SE and UEA in term of reducing ability. Moreover, the crude extraction yield obtained with SC-CO_2_ extraction was significantly higher than that reported by using UAE. It was found that the extract obtained by SC-CO_2_ extraction is a rich source of linoleic acid (18:2) compared with that obtained by SE, so it can provide new natural products to the food industry. Thus, it can be concluded that *Benincasa hispida* seed is a potential source of valuable compounds and the green SC-CO_2_ extraction technique is an effective and promising process to obtain *Benincasa hispida* seeds oil with strong antioxidant activity and also rich in essential fatty acids.

## Figures and Tables

**Table 1:** Experimental design (coded and uncoded levels) and results of response variable.

Run	Block	Process variables			CEY (mg-extract/g-dried sample)
		Pressure (bar)	Temperature (°C)	Dynamic extraction time (min)	
1	1	271 (+1)	48 (+1)	72 (−1)	159.24
2	1	271 (+1)	48 (+1)	108 (+1)	161.88
3 (C_p_)	1	225 (0)	45 (0)	90 (0)	172.43
4 (C_p_)	1	225 (0)	45 (0)	90 (0)	172.85
5	1	179 (-1)	48 (+1)	72 (−1)	132.73
6	1	179 (-1)	42(−1)	108 (+1)	128.18
7 (C_p_)	1	225 (0)	45 (0)	90 (0)	173.22
8	1	179 (-1)	42 (−1)	72 (−1)	125.62
9	1	179 (-1)	48 (+1)	108 (+1)	140.00
10 (C_p_)	1	225 (0)	45 (0)	90 (0)	171.87
11	1	271 (+1)	42 (−1)	72 (−1)	161.12
12	1	271 (+1)	42 (−1)	108 (+1)	161.20
13	2	225 (0)	45 (0)	120 (+1.63)	173.10
14 (C_p_)	2	225 (0)	45 (0)	90 (0)	173.33
15	2	225 (0)	50 (+1.63)	90 (0)	137.50
16 (C_p_)	2	225 (0)	45 (0)	90 (0)	173.87
17	2	150 (−1.63)	45 (0)	90 (0)	119.11
18	2	225 (0)	40 (−1.63)	90 (0)	127.00
19	2	300 (+1.63)	45 (0)	90 (0)	168.53
20	2	225 (0)	45 (0)	60 (−1.63)	166.70

(C_p_), Centre point.

**Table 2:** Analysis of variance (ANOVA) and coefficients of the final reduced regression equation.

Source	df ^a^	CEY(mg-extract/g-dried sample)		
		Coefficient	Sum of squares	*p*-Value
Model	9	172.88	7,392.83	<0.0001
X_1_	1	14.82	2,928.80	<0.0001
X_2_	1	2.62	91.23	<0.0001
X_3_	1	1.73	39.68	0.0001
X_1_^2^	1	−10.77	1,532.34	<0.0001
X_2_^2^	1	−15.11	3,015.37	<0.0001
X_3_^2^	1	−0.99	12.99	0.0052
X_1_X_2_	1	−2.52	50.65	<0.0001
X_1_X_3_	1	−0.89	6.32	0.0307
X_2_X_3_	1	0.91	6.61	0.0280
Residual	9		8.68	
Lack of fit	5		7.53	0.0673
Pure error	4		1.15	
Total	19		7,401.60	
R^2^		0.998		
Adjusted- R^2^		0.997		
C.V.%		0.630		
E (%)		0.350		

^a^ Degree of freedom.

**Table 3:** Comparison between different extraction methods.

Extraction Mode	CEY (mg-extract/g-dried sample)	Antioxidant activity		Fatty acid composition *								
		%DPPHsc	%ABTSsc	C14:00 ^a^	C16:00 ^b^	C16:01 ^c^	C18:00 ^d^	C18:01 ^e^	C18:02 ^f^	C18:03 ^g^	ΣSFA ^h^	ΣUFA ^i^
CSE (EtOH, 99.5%)	250.00 ± 1.30	28.70 ± 0.70	27.00 ± 0.90	1.60 ± 0.11	15.30 ± 0.15	0.68 ± 0.27	7.40 ± 0.18	14.10 ± 0.15	60.60 ± 0.13	-	24.30	75.38
UAE (EtOH, 99.5%)	108.62 ± 0.78	35.84 ± 0.42	43.10 ± 0.63	1.30 ± 0.11	10.80 ± 0.25	0.80 ± 0.33	5.70 ± 0.14	14.40 ± 0.22	66.20 ± 0.10	0.60 ± 0.12	17.80	82.00
SCE (SC-CO_2_+EtOH)	175.60 ± 0.33	53.20 ± 0.54	62.22 ± 0.25	1.07 ± 0.18	9.83 ± 0.33	0.95 ± 0.13	5.07 ± 0.21	14.73 ± 0.10	67.17 ± 0.15	0.68 ± 0.12	15.97	83.53

* The results of fatty acids are expressed as % of total fatty acids; ^a^ Myristic acid; ^b^ Palmitic acid; ^c^ Palmitoleic acid; ^d^ Stearic acid; ^e^ Oleic acid; ^f^ Linoleic acid; ^g^ α-linolenic acid; ^h^ Total saturated fatty acid; ^i^ Total unsaturated fatty acid.
